# Case report of coronary artery fistula: Added value of multimodal advanced imaging with cardiac computed tomography and cardiac magnetic resonance

**DOI:** 10.1097/MD.0000000000018255

**Published:** 2019-12-10

**Authors:** Bruna Punzo, Ernesto Forte, Marco Salvatore, Carlo Cavaliere, Filippo Cademartiri

**Affiliations:** IRCCS SDN, Naples, Italy.

**Keywords:** cardiac computed tomography, cardiac magnetic resonance, coronary artery fistula, dual source computed tomography

## Abstract

**Rationale:**

Unlike invasive coronary angiography and echocardiography, cardiac computed tomography (CCT) and cardiac magnetic resonance (CMR) imaging allow a coronary artery fistula (CAF) comprehensive evaluation focusing on both coronary and myocardial findings.

**Patient concerns:**

We present the case of an asymptomatic patient suspected for CAF and referred to our structure for cardiovascular evaluation.

**Diagnosis:**

The patient was diagnosed a CAF without coronary artery disease on the basis of CMR and CCT.

**Interventions:**

The patient underwent an invasive coronary angiography after which the medical staff decided not to surgically treat the CAF. Therefore, a conservative treatment was chosen with strict temporal monitoring.

**Outcomes:**

After less than 1 year follow-up, the patient presented stable conditions without complaints.

**Lessons:**

Multimodal non-invasive imaging has a key role in patient assessment for disease diagnosis providing better understanding for prognosis and treatment.

## Abstract 2

Supplemental Digital Content is available in the text

## 1 Introduction

Coronary artery fistulas (CAFs) are coronary anomalies incidentally detected in asymptomatic patients. Among imaging modalities, ECG and chest x-ray are routinely performed but have minimum benefit to ascertain the diagnosis. Echocardiography is widely used to detect congenital and acquired heart defects, as coronary dilation, even if it is sometimes difficult to deal with the detail anatomy and some inaccuracy can occur when calculating coronary flow for shunt assessment.^[1]^ Many literatures report the key role of invasive coronary angiography in CAF assessment.^[2]^ This modality could not provide a clear fistula visualization due to contrast medium dissipation in high-pressure vessels and should be recommended only for patient treatment.^[3]^ Undoubtedly, Cardiac Computed Tomography (CCT) has a great diagnostic value in coronary visualization thanks to its high spatial resolution and is widely used for coronary anomalies detection.^[4]^ Despite the limited role of Cardiac Magnetic Resonance (CMR) in coronary assessment, it should be underlined that it has unique capabilities in flow quantification by phase contrast sequences as well as myocardial tissue characterization.^[5]^

We present the case of a 50-years-old asymptomatic man who performed both CCT and CMR for coronary artery fistula investigation.

## 2 Case presentation

A 50-years-old asymptomatic male was referred to our institution to perform a CMR. A previous echocardiography, performed in the context of a general check-up, was suggestive for suspicion of dilated coronary sinus and a rest ECG presented overload signs and atypical electrical recovery. Cardiovascular risk factors, including familiarity for atherosclerotic coronary artery disease, smoking and obesity, were recorded. The patient was not under any pharmacological treatment and baseline laboratory parameters were within normal limits (CRP < 0.50 mg/L; ESR: 9 mm; Glu: 73 mg/dL; Cr: 0.83 mg/dL; TC: 180 md/dL; HDL: 56 mg/dL; LDL: 118 mg/dL; TGL: 66 mg/dL; Hcy: 19.7 μmol/L).

He underwent CMR on a 1.5 T scanner (Achieva dSTREAM 1.5 T, Philips) with a 32 channels body coil. Morphological and functional sequences T2/short tau inversion recovery (STIR)/Cine steady state free precession (SSFP) in main cardiac planes and phase contrast sequences in both aortic and pulmonary valve plane were acquired. Magnetic resonance images of the coronary vessels were obtained by a 3D balanced respiratory navigated SSFP sequence with fat saturation and T2 pre-pulse (echo time = 2–3 ms, repetition time = 4–6 ms, matrix = 480 × 480). Quantitative analysis to calculate Qp/Qs, ejection fraction (EF), end-diastolic volume (EDV), end-systolic volume (ESV), was performed by advanced dedicated workstations (CVI42, Circle, Canada; Syngo.Via VB10B, Siemens). Based on the findings of CMR the patient was directly referred for CCT.

CCT was performed with a 3rd generation dual source multidetector scanner (DSCT, Somatom Force, Siemens Healthineers, Erlangen, Germany). At first, a non-contrast CT prospectively ECG-triggered high pitch spiral acquisition (FLASH) was performed for calcium score evaluation. Afterwards, an angiographic CT scan with retrospective ECG gating and automated attenuation-based anatomical tube current modulation (CARE Dose 4D, Siemens) was performed. Tube voltage was adjusted by the use of an automated attenuation-based tube voltage selection functionality (CARE kV, Siemens). For the angiographic scan, 70 mL of iodinated contrast agent (Iomeprol 400mgI/mL, Iomeron 400, Bracco, Italy) at 5.5 mL/s followed by 50 mL of saline at the same flow were injected. Data were reconstructed by a dedicated 3rd generation advanced modelled iterative reconstruction (ADMIRE, Siemens Healthineers, Erlangen, Germany) using medium sharp convolution kernels (Bv36 and Bv40), strength level of 3, section thickness of 0.75 mm with an increment of 0.4 mm and pixel matrix of 512 × 512. Post processing was performed with a dedicated workstation (Syngo.Via VB10B, Siemens Healthineers, Erlangen, Germany) and MIP, c-MPR,3D volume and cinematic rendering (VR and CR, respectively) images were generated.

**Figure 1:**
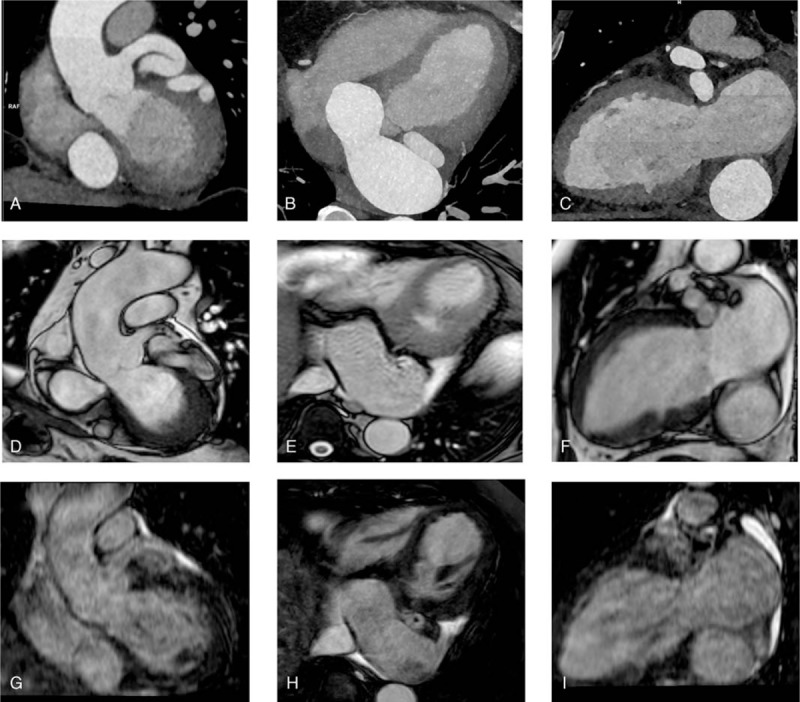
(A-D-G) Cardiac computed tomography (CCT) multiplanar reformation (MPR), cardiac magnetic resonance (CMR) three-chamber view and CMR SSFP MPR representing the origin of the fistula. (B-E-H) CCT MPR, CMR four-chamber view and CMR SSFP MPR showing the enlarged coronary sinus. (C-F-I) CCT MPR, CMR two-chamber view and CMR SSFP MPR where proximal and distal left circumflex coronary artery and the coronary sinus appear both dilated.

**Figure 2:**
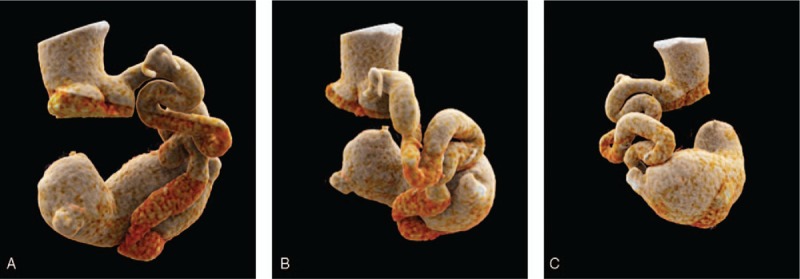
(A-B-C) Cinematic rendering (CR) of the coronary artery fistula (CAF): the origin, the tortuous course and the drainage site into the coronary sinus are well appreciated.

CMR analysis showed a biventricular dilatation (Left Ventricle EDV = 136 mL/m^2^, Right Ventricle EDV = 117 mL/m^2^) with preserved EF (69%). A dilated left circumflex coronary artery (maximum diameter = 15 mm), with a high likelihood of draining into the coronary sinus was detected. Evidence of a significant left to right shunt (Qp/Qs = 1.4) was present. The suspicion of a coronary artery fistula was raised. However, it was not possible to properly display the site of fistulation. CCT confirmed the fistula: the circumflex coronary artery (LCX) originated from the left main coronary artery (maximum diameter 15 mm; that is, significantly dilated) and appeared tortuous and diffusely dilated along the entire left atrio-ventricular groove (maximum diameter 15 mm), with no stenosis or atheromatous disease (Coronary Calcium according to Agatston score was 0). It terminated into a large fistulous plexus connected to coronary sinus, located at 7 cm from the outlet in the right atrium that, in this tract, showed a diameter of 4 cm (Figs. 1 and 2).

**Figure 3:**
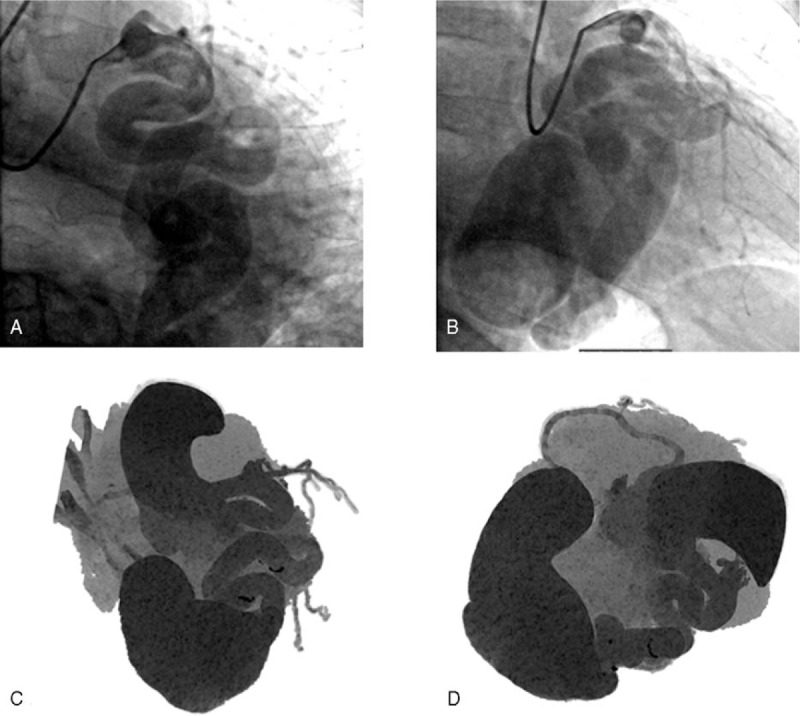
(A–B) Invasive coronary angiography and (C–D**)** Maximum intensity projection (MIP) cardiac computed tomography (CCT) images representing the fistulous tract.

Subsequently, 7 months later, the medical team decided to refer the patient to a coronary angiography in view of a possible treatment. The examination confirmed the presence of a voluminous CAF draining into the coronary sinus (Fig. 3) but the surgery staff addressed the patient to a strict temporal monitoring. Currently (less than 1 year), the patient is in follow-up and presents stable conditions without complaints.

## 3 Discussion

A CAF is a congenital or acquired abnormal vascular communication of coronary arteries with cardiac chambers or any segment of the systemic or pulmonary circulation, without an intervening capillary network.^[6]^

CAFs can be classified according to their origin, drainage site, or complexity. The right coronary artery (RCA) is the most common origin site of CAFs accounting for 50% to 55% of cases. The left anterior descending artery (LAD) is involved in about 35% to 40% of cases, while the LCX, as for the reported case, for the 5% to 20%.^[6,7]^ Moreover, the most common drainage site is represented by the pulmonary trunk (89%), followed by the right ventricle (41%), the right atrium (26%), the left atrium-left ventricle (3%–5%) and the coronary sinus (7%), as the case described.^[8]^ Even though most patients are asymptomatic, in some cases they present atypical or typical signs and symptoms such as right ventricular enlargement, cardiac dysfunction, endocarditis and arrhythmias, dyspnea, orthopnea, and chest pain.^[9]^ From a pathophysiological point of view, a dilated and tortuous CAF could change the normal course of myocardial blood flow causing its reduction and, in some cases, ischemia in downstream myocardial territories. Subsequently, the fistulous coronary artery can undergo a compensatory mechanism driven by the pressure gradient existing between the 2 communicating structures and leading to coronary enlargement or notable tortuous course, with several possible complications such as aneurysm development, atherosclerotic deposition, and even rupture in a few cases.^[3,10,11]^ Another consequence of a CAF is left to right shunt with progressive dilatation of right and left ventricle, depending on the anatomical size, location, flow rate, and off course duration over time.

The reference standard imaging technique for CAF assessment has been for decades invasive coronary angiography that allows diagnosis. Moreover, the anatomical relationships with the surrounding structures could be poorly appreciated and, especially when CAFs are very large and/or they have a very high flow rate, the course of the fistula can be underestimated or difficult to properly visualize. In our case, the patient performed a conventional coronary angiography but the surgical team decided not to proceed to surgery.

Technological developments in the field of CMR and CCT have led to a more accurate anatomical and functional assessment. CMR and CCT are useful to assess coronary morphology, cardiac function, myocardial perfusion and tissue characterization, in particular for patients in absence of symptoms where a rigorous and periodic follow-up is recommended.

CMR, thanks to its high contrast resolution, allows multiparametric myocardial tissue characterization. In detail, T2 weighted sequences with and without fat suppression are used to visualize inflammation and edema while SSFP sequences are widely applied for vascular examinations and the assessment of cardiac kinetic and function. A comprehensive scan protocol should include delayed enhancement sequences acquired 8 to 10 minutes after IV administration of a gadolinium-based contrast agent with inversion time set in order to null the healthy myocardium. Flow related information can be extracted by phase contrast sequences whose employment is of particular relevance in case of shunt suspicion.^[12]^

Un-enhanced coronary magnetic resonance angiography has been allowed by 3D balanced respiratory navigated sequences with fat suppression and T2 pulse preparation. This is a promising tool for vessel visualization without the need of contrast agent administration. Moreover, 3D acquisition provides an easy post-processing together with higher signal to noise ratio and spatial resolution. Nevertheless, it is affected by less in-flow effects, it is time consuming and limited in distal coronary definition.^[13]^

In our case, CMR has highlighted the presence of a significant biventricular dilation with preserved global systolic function. Furthermore, the coronary fistula arising from the left circumflex artery and draining into the coronary sinus with evidence of left to right shunt has been well delineated and quantified. CCT, despite the use of ionizing radiations, has provided a better anatomical definition than CMR, depicting with greater accuracy the fistulous tract and the exact drainage site.

Among CCT data reconstruction algorithms, CR is a new method of 3D visualization for volumetric data^[14]^ that renders clinical images more adherent to human anatomy. Although CR is still a developing technique, it could be a valuable treatment planning tool for both specialists and vascular surgeons. Additionally, as radiology strives to become a more patient-centered field of medicine, the quality of these images can facilitate explanation of disease state to patients (Supplemental video, (Cinematic rendering (CR) showing the origin, the course and the drainage site of the coronary artery fistula (CAF).)).

## 4 Conclusion

In conclusion, CCT and CMR are useful for non-invasive CAF detection, highlighting its origin and course, and providing functional and morphological information essential for patient prognosis. In particular, in asymptomatic patients a strict temporal monitoring is needed and a detailed cardiovascular examination cannot be evaded.

## Author contributions

**Methodology:** Bruna Punzo.

**Supervision:** Marco Salvatore, Carlo Cavaliere, Filippo Cademartiri.

**Visualization:** Ernesto Forte.

**Writing – original draft:** Bruna Punzo.

**Writing – review & editing:** Bruna Punzo.

## Supplementary Material

Supplemental Digital Content
